# A stressful life (or death): Combinatorial proteotoxic approaches to cancer-selective therapeutic vulnerability

**DOI:** 10.18632/oncotarget.266

**Published:** 2011-04-21

**Authors:** Paul Workman, Faith E. Davies

**Affiliations:** ^1^ Signal Transduction and Molecular Pharmacology Team, Cancer Research UK Cancer Therapeutics Unit, Division of Cancer Therapeutics, The Institute of Cancer Research, Haddow Laboratories, 15 Cotswold Road, Sutton, Surrey SM2 5NG UK; ^2^ Myeloma Targeted Treatment Team, Divisions of Molecular Pathology, Clinical Studies & Cancer Therapeutics, Institute of Cancer Research, Brookes Lawley Building, 15 Cotswold Road, Sutton, Surrey SM2 5NG UK

Maintaining protein homeostasis within a cell is vital. Recent studies have suggested that therapeutically manipulating intracellular protein handling pathways in cancer cells perturbs protein homeostasis and results in the delivery of a novel apoptotic signal.

A new paper in *Oncotarget* by Neznanov *et al* [1] reinforces the potential of proteotoxic stress-targeted therapy and in particular demonstrates the ability of combinatorial approaches to enhance the antitumor effects of the proteasome inhibitor bortezomib by induction of protein misfolding using hyperthermia or the antibiotic puromycin. In particular, the new results illustrate the therapeutic potential of combining non-toxic doses of puromycin with bortezomib in a mouse model of multiple myeloma.

There are a number of potential ways of perturbing protein homeostasis: firstly by forcing the apoptotic signal by disturbing protein quality control with the premature degradation of key growth and survival molecules; secondly by inhibiting the degradation of proteins resulting in a build up of unwanted proteins; or finally by interfering with key protein folding pathways resulting in the build-up of misfolded proteins. The end result of each of these processes is programmed cell death. Crucially, malignant cells are more susceptible to killing through the manipulation of proteostasis, resulting in a cancer-selective vulnerability.

**Figure 1:**
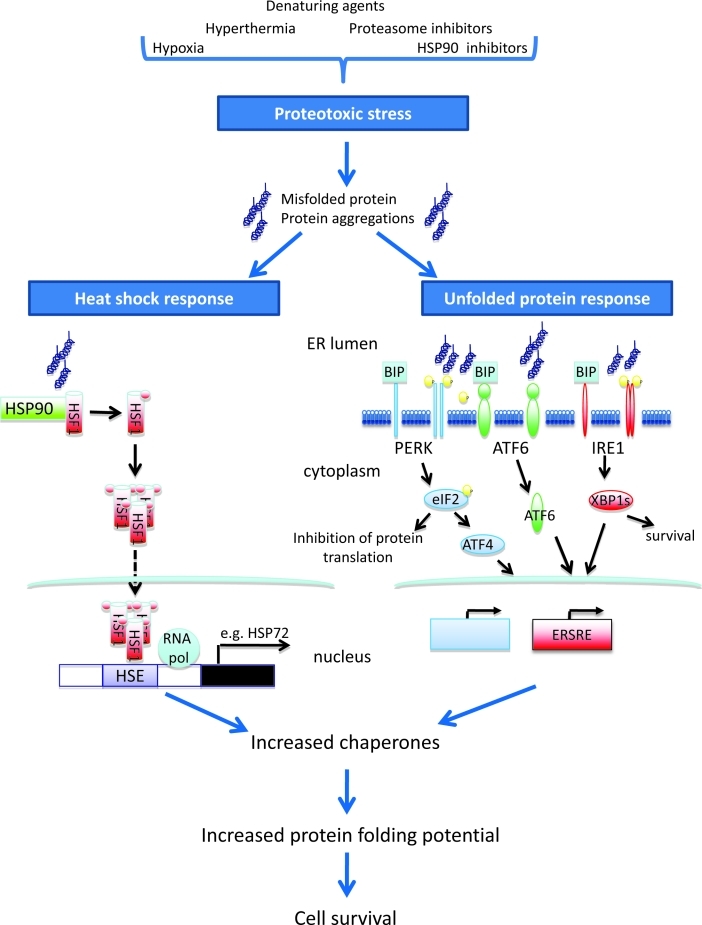
Vulnerable proteotoxic stress pathways for exploitation in cancer-selective combinatorial therapies

The build up of proteins that have failed to fold correctly results in the presence of non-functional proteins, a tendency toward protein aggregation and impaired cellular function and is referred to as proteotoxic stress (PS). A cell placed under such stress has two possible physiological responses. Initially it will resist death whilst attempts at correct protein folding are carried out. However, if this fails then an apoptotic signal is delivered. Two highly conserved systems are in place to combat PS – the unfolded protein response (UPR) and the heat shock response (HSR). Both systems act as quality control processes ensuring the correct folding and 3D conformation for functionally active proteins. The UPR senses unfolded native proteins within the endoplasmic reticulum (ER) and ensures their correct folding, processing, export or degradation. Activation of the UPR results in a bias of protein translation towards the synthesis of chaperone proteins involved in protein folding within the ER, an increase in disposal of misfolded proteins via the ubiquitin proteasome pathway, and the delivery of a survival signal. If the build-up of misfolded protein is irreversible, the cell undergoes apoptosis [2,3]. The HSR is activated by the accumulation of non-native proteins in the cytosol or nucleus. Once activated there is an increase in the synthesis of molecular chaperones that both facilitate protein folding and also suppress protein aggregation. In addition, the heat shock proteins also have a broader anti-apoptotic role mediating both the intrinsic mitochondrial-dependent and extrinsic death receptor-dependent apoptotic pathways [4,5]. The balance, therefore, between the induction of proteotoxic stress and the adaptive UPR and HSR is vital for protein homeostasis and cell survival (Figure 1).

A number of studies have demonstrated that cancer cells have intrinsically high levels of PS. This is a result of the accumulation of misfolded proteins caused by cancer cells surviving within an unfavourable hypoxic micro-environment, as well as an increase in protein misfolding resulting from aneuploidy and the expression of mutated or over-abundant oncogenic proteins, especially in highly secretory tumours. The increased level of PS results in a dependence of the cancer cell on both the UPR and HSR to maintain protein homeostasis and allow survival in the stressed malignant state. Studies have demonstrated that cancer cells overexpress heat shock protein family members [4,5] and have high levels of a number of components of the UPR pathway (e.g. XBP1s) [6] which aid cell survival and mediate drug resistance.

Over recent years a number of investigators have attempted to alter the balance between PS, the UPR and the HSR to induce cancer cell death. The clinically most advanced of these approaches involve the induction of PS using either proteasome or heat shock protein 90 (HSP90) inhibition. The ubiquitin proteasome pathway is responsible for the degradation of many key cellular signaling proteins. In multiple myeloma, where the use of proteasome inhibitors is considered standard of care, both in-vitro data and clinical studies have demonstrated that inhibition of the catalytic subunit of the 20S proteasome by bortezomib results in a build up of unwanted proteins, the induction of cellular stress and apoptosis [7,8]. Similar results have been seen both in-vitro and in-vivo in solid and hematological malignancies following HSP90 inhibition [9,10,11]. HSP90 is a crucial chaperone protein, ensuring correct folding and activation of its clients, which if not correctly folded are targeted for degradation by the proteasome. Many of HSP90's client proteins are key tumor growth and survival signaling molecules such as EGFR, AKT, BRAF, CRAF, CDK4 and mutant p53. HSP90 inhibition results in a decrease in survival molecules, thus sensitizing cells to apoptosis, as well as preventing correct protein folding leading to increased PS.

In addition to inducing PS, a number of investigators have explored approaches aimed at inhibiting the HSR. The heat shock protein 70 family (HSP70) is a major mediator of the HSR. Heat-shock inducible HSP70 (HSP72) plays a key role in protecting cells from stress by reducing stress-induced protein aggregations and thus protecting cells from apoptosis. It also interacts with members of the intrinsic and extrinsic apoptotic pathways and p53, as well as being a co-chaperone for HSP90. The expression of HSP70 family members is tightly controlled by the transcription factor, heat shock factor 1 (HSF1). In response to stress, including HSP90 inhibition, HSF1 dissociates from the HSP90/HSP72 complex and translocates to the nucleus where it initiates transcription of protective stress response chaperones, reducing damaged protein aggregations and facilitating degradation by the proteasome. The constitutively expressed heat shock cognate 70 (HSC70), another HSP70 family member, also plays a significant anti-apoptotic role. Our own studies have shown that silencing HSC70 or HSP72 alone has no effect on tumor cell death, whereas simultaneously reducing the expression of both of these isoforms induces degradation of HSP90 clients, G1 arrest and apoptosis [12,13]. Others have shown similar results with HSF1 silencing [14,15].

A number of groups have explored a combination of the above approaches – targeting PS and HSR simultaneously. Proteasome or HSP90 inhibition results in activation of the HSR via HSF1 driving upregulation of HSP70 family members [3,10,16]. Using siRNA we have shown increased susceptibility to HSP90 inhibitors when HSP72 and HSC70 are targeted simultaneously [12,16]. Therefore, combinations involving either the proteasome or HSP90 inhibition with HSP70 targeting offers an attractive way to potentiate single agent therapeutic activity.

Neznanov *et al* present a further approach to manipulating the balance between PS and HSR [1]. Rather than inhibiting the HSR, they dramatically increase PS by both inducing protein misfolding and inhibiting protein degradation via the proteasome, so-called ‘enhanced proteotoxic stress’. They demonstrate that the increased level of misfolded proteins, induced by either hyperthermia or puromycin (which works by causing premature termination of translation and accumulation of aborted and incorrectly folded products) in combination with bortezomib, could not be matched by the cancer cell's ability to synthesize chaperones as part of the HSR and thus cell death ensued. The studies indicate that the enhanced proteotoxic death is, at least in part, mediated through p53. This raises questions as to the effect of p53 status in tumor versus healthy cells on the response to combinatorial treatments proposed. Importantly, however, the synergistic anticancer effect was seen both in-vitro and an in-vivo mouse model of multiple myeloma and thus provides a rationale for attempting such an approach or other PS combinations in the clinical arena.

A wealth of data now supports various tactics to modulate protein homeostasis as an approach to cancer-selective therapy. The stressed state of malignant cells leaves them vulnerable to therapeutic strategies that target molecular determinants of the UPR or the HSR – or a combination of the two – as orthogonal approaches to exacerbate enhanced PS. Indeed stress phenotypes, including PS, can now be included among the extended hallmarks of cancer – in addition to the Hanahan and Weinberg traits [17]. Furthermore, proteotoxic stress targets can be considered as part of a broader conceptual framework in which non-oncogene targets as well as oncogene targets can be attacked in tumour-selective therapy [17].
